# Racial and ethnic inequities to noise pollution from transportation- and work-related sources in the United States

**DOI:** 10.1038/s41370-025-00795-x

**Published:** 2025-07-17

**Authors:** Abas Shkembi, Keshav Patel, Lauren M. Smith, Helen C. S. Meier, Richard L. Neitzel

**Affiliations:** 1 Department of Environmental Health Sciences, University of Michigan School of Public Health, Ann Arbor, MI USA; 2 Survey Research Center, University of Michigan, Ann Arbor, MI USA

**Keywords:** Cumulative impacts, Environmental justice, Occupational justice, Noise mapping, Geospatial analysis

## Abstract

**Background:**

Racial and ethnic inequities in environmental noise exist in the US, partially attributable to historical structural racism. However, previous studies have not considered the totality of people’s exposures. Since people spend most of their waking time at work, there is a need to consider cumulative exposure to noise both in and out of the workplace to understand who is most at risk of noise pollution-related adverse health outcomes.

**Objectives:**

To (1) investigate whether racial and ethnic minority communities are disproportionately burdened by transportation- and workplace-related noise pollution, and (2) assess whether structural racism through historically redlined neighborhoods with sustained mortgage discrimination partially contribute to the hypothesized inequity.

**Methods:**

We characterized the prevalence of workplace noise and transportation noise exposure by census tract across the US. We analyzed the census tract-level association between racial and ethnic composition and the population exposed to both transportation- and workplace-related noise pollution in the 2010s using geospatial models. We then assessed census tract-level associations with transportation and workplace noise pollution using historical redlining in the 1930s as the primary covariate, stratified by mortgage discrimination in the 1990s using a similar geospatial model, controlling for census tract-level indicators of low socioeconomic status.

**Results:**

Higher percentages of racial and ethnic minority individuals, particularly Hispanic/Latino and non-Hispanic Black Americans, were associated with significantly higher odds of exposure to both transportation and workplace noise (odds ratio = 8.59, 95% CI: 7.38–10.0, when comparing within-metropolitan area, highest to lowest quintile percentages). These disparities are particularly profound in urban areas. Urban tracts which experienced residential segregation in the 1930s, even without sustained mortgage discrimination in the 1990s, have a significantly higher percentage of individuals exposed to both transportation and workplace noise today compared to those without historical segregation (1.55%, 95% CI: 1.37–1.74). This inequity is even higher among historically segregated tracts that experienced sustained mortgage discrimination (1.83%, 95% CI: 1.66–2.01).

**Significance:**

These findings can advance environmental justice initiatives by informing regulatory action to protect communities of color from noise pollution both environmentally and during work.

**Impact:**

Our study provides evidence that neighborhoods with a higher proportion of racial and ethnic minority individuals are cumulatively burdened by noise pollution both during work and from transportation sources in their home communities. This suggests that not incorporating workplace exposures when assessing environmental impacts may overlook the most burdened communities. Future environmental justice efforts and policies should consider assessing workplace exposures to reduce environmental health disparities more effectively.

## Introduction

Despite large reductions in air and water pollutant levels across the US over the past several decades, noise pollution continues to impact more than 100 million Americans [1], particularly racial and ethnic minority individuals [2]. Transportation-related noise, a contributor to cardiovascular disease, hearing loss, and cognitive impairment, is a significant source of environmental noise pollution in urban areas [3]. However, most adults spend a third of their time at work and studies of noise pollution typically do not consider workplace-related noise together with transportation-related noise. Ignoring the time people are exposed to noise during work may underestimate racial and ethnic inequities to noise pollution since racial and ethnic minority workers are disproportionately employed in typically noisy occupations/industries, such as construction [4].

The fragmented approach of investigating environmental and workplace noise separately is in part driven by ineffective noise regulations in the US. The US Environmental Protection Agency (EPA) is tasked with regulating environmental sources of noise pollution since the passage of the 1972 Noise Control Act, which the EPA’s Office of Noise Abatement and Control (ONAC) enforced for nine years. However, a complete defunding of ONAC in 1981 has effectively prevented the EPA from reducing ambient noise pollution in the US [5]. This has left nearly one in three Americans likely exposed to daily ambient noise pollution above 70 dBA as of 2014 [1]—a daily level that may cause noise-induced hearing loss over a lifetime according to the EPA [6]. The World Health Organization recognizes that levels as low as 55 dBA may cause cardiovascular disease [3], suggesting that even more than one in three Americans may be burdened by excessive noise pollution. While other federal agencies (e.g., the Department of Transportation) and local zoning laws, ordinances, and urban planning developments consider excessive noise pollution in their decision making, only the EPA has a mandate from Congress to coordinate all federal efforts to control excessive community noise pollution in the US. In workplaces, however, the US Occupational Safety and Health Administration (OSHA) has been regulating occupational noise since 1971. Still, millions of workers continue to be exposed to hazardous levels (>85 dBA over 8 h) [7–9], a level known to cause material hearing loss in as many as 1 in 3 exposed workers and linked to cardiovascular disease and injuries [10]. The EPA’s failure to regulate environmental noise pollution (e.g., from transportation) and OSHA’s insufficiently protective regulation of workplace noise has likely created a situation in the US where people in some communities are excessively exposed to both environmental and workplace sources of noise pollution; however, the extent of these cumulative overexposures is unknown.

**Fig. 1:**
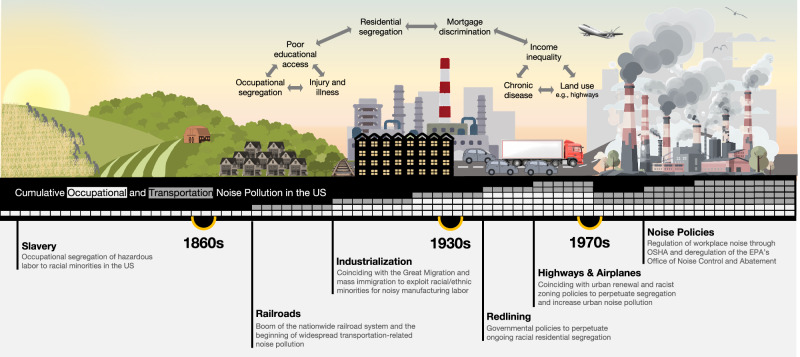
An illustration depicting the history of changing occupational and environmental landscapes from pre-1860s to today which influenced occupational- and transportation-related noise pollution in the US. The middle banner represents how cumulative occupational (in light gray) and transportation (in dark gray) noise pollution likely increased as a result of major changes to the workplace and cities, with more boxes indicating higher cumulative noise pollution. Icons in the figure were downloaded from PowerPoint, https://purepng.com/, and https://favpng.com/.

We hypothesize that structural racism may contribute cumulative noise inequities, as we’ve illustrated in the conceptual model in Fig. 1 with a backdrop of changing occupational and environmental landscapes that influenced noise pollution over the last two centuries. The enslavement of Black Americans and indentured servitude of immigrants laid the foundation in the US for occupationally segregating racial and ethnic minority workers to more hazardous labor. As consequence of mass industrialization and migration, Black Americans and immigrants were exploited to work in noisy, industrial jobs in the early 1900s, like in Michigan [11, 12]. Around the same time institutional strategies to racialize space, including racially restrictive covenants and historical redlining used both federal and local laws, kept racial and ethnic minority families residentially segregated from White families [13]. This laid the groundwork for racist land use and urban renewal policies in the 1950s that resulted in minority communities in urban areas being burdened—and in some cases destroyed—by highways and airports [14, 15], substantially increasing transportation-related noise inequities in the US. Continued disinvestment from mortgage discrimination deepened income inequality by limiting the property tax base of minority homes [16], directly influencing educational access and employment opportunities that have likely resulted in occupational segregation of racial and ethnic minority individuals into noisier jobs. As a consequence, many of the same minority communities that face disproportionately higher transportation-related noise pollution may be simultaneously burdened by excessive and harmful workplace-related noise, potentially creating a situation of cumulative noise inequities in the US today that has not yet been explored.

Previously, nationwide studies evaluating associations of both high transportation- and work-related noise pollution were not possible because nationwide estimates of workplace noise exposure were not available at a fine geographic scale. However, our team recently developed a method to estimate workplace noise pollution at the census tract-level across the US [17] which can be merged with publicly available data on transportation-related noise pollution. In this study, our main objectives were to (i) characterize the prevalence of workplace- and transportation-related noise pollution across the US, (ii) assess whether a higher proportion of racial and ethnic minority individuals are also those likely to have high exposure to both workplace- and transportation-related noise, and (iii) investigate whether structural racism through historically redlined neighborhoods with sustained mortgage discrimination would be inequitably exposed to cumulative noise today.

## Methods

### Census tract-level transportation and workplace noise pollution prevalence

To estimate census-tract level transportation noise pollution, we developed population-weighted estimates of the percentage of individuals exposed to 24-hour noise (L_Aeq_ in A-weighted decibels, dBA) > 55 dBA, a level considered hazardous to human health for all sources of transportation noise [3], from road, rail, and air transportation sources for each census tract in the US. These estimates are based on transportation noise estimates from the 2018 Department of Transportation (DOT) National Transportation Noise Map (NTNM) [18] using a Monte Carlo simulation approach that aggregates estimates from the census block level (*n* ~ 8,000,000) up to to the census tract level (*n* ~ 70,000). This reduces the probability of overestimating the number of individuals exposed when aggregated up to the census tract level. We excluded estimates with ≤20 residents. See Appendix A in the Supplemental Materials for further details.

To estimate census-tract level workplace noise pollution, we developed estimates for the percentage of workers exposed to >85 dBA, a level considered hazardous [10], of workplace noise for each census tract in the US. These estimates have been previously developed by our team [17]. We applied a Monte Carlo simulation approach to characterize exposure to hazardous workplace noise for every census tract. To achieve this, we integrated information on (i) the types of jobs people do and where, (ii) the typical noise exposures of major occupational groups and (iii) whether these exposures meet the US National Institute for Occupational Safety and Health (NIOSH) Recommended Exposure Limit (REL) for likely hazardous noise exposure. We answered these questions by leveraging (i) employment counts by major occupational groups from the US American Community Survey at the census tract level, (ii) >1 million estimates of 8-hr, time weighted average noise exposure by major occupational groups from a nationwide noise job exposure matrix (NoiseJEM) [19] and a previously published hierarchical Bayesian model [20], and (iii) whether these estimates were above the NIOSH REL of 85 A-weighted decibels. We excluded estimates with ≤20 workers. See Appendix B in the Supplemental Materials for further details.

To identify census tracts that were burdened by cumulatively high workplace and transportation noise, we identified whether the census tract was among the >75th percentile of both workplace and transportation noise pollution across the US. The code for simulation and final data for the transportation and workplace noise pollution prevalences by census tract are available at https://github.com/abasshkembi/noise-oej.

### Census tract-level racial and ethnic composition and other sociodemographic information

Census tract-level racial and ethnic composition variables were derived from the 2015–2019 American Community Survey (derived from the “tidycensus” R package) [21] as the proportion of non-Hispanic residents who are White, Black, Asian, American Indian or Alaskan, or Native Hawaiian or Pacific Islander, as well as the proportion of Hispanic Americans. Racial and ethnic minority individuals are considered those who are not non-Hispanic White. We incorporated three indicators of socioeconomic status from the 2015–2019 American Community Survey (commonly characterized by factors related to money, education, and employment [22]): the proportion of residents who are low income (reflecting money and defined as those below 200% of the federal poverty level); those without a high school diploma (reflecting education); and the proportion of unemployed individuals (reflecting employment) to account for the non-working adult population within a census tract. Census tracts were defined as urban or non-urban using the US Department of Agriculture Rural-Urban Commuting Area Codes [23], where a level of 1–3 (metropolitan) were considered “urban” and levels 4–10 (micropolitan, small town, and rural) were considered “non-urban”.

### 1930s historical redlining and 1990s mortgage discrimination

We used historical redlining scores for 2010 census tract boundaries since original neighborhood boundaries outlined in 1930s Home Owners’ Loan Association (HOLC) maps do not overlap with current-day census tracts [24]. The historical redlining score is an area-weighted weighted calculation of HOLC grades (A to D, with D being considered historically redlined) for each census tract with at least 20% overlap with an HOLC-defined neighborhood. These census tracts are assigned a score between 1 and 4, with higher scores indicating worse grades (a greater proportion of D and C grades). This weighted score approach has been shown to have the highest sensitivity in correctly classifying HOLC grades compared to other approaches, although it may mask qualitative differences in HOLC grades and has low specificity [25]. This approach has also been used previously in other environmental justice studies of redlining [26–30].

Mortgage discrimination in the 1990s comes from the Home Mortgage Disclosure Act Longitudinal Dataset by census tract (HLD), which spatially and temporally harmonized mortgage lending data since 1981 [31], and data on household counts by race and ethnicity from the 2000 decennial Census. We used data from 1990 to 2000 because we assumed that the mechanisms by which mortgage discrimination impacts transportation- and workplace-related noise pollution (e.g., income inequality, poor educational access) may be lagged by a few decades (the noise pollution data are estimates from the 2010s). We characterized racial and ethnic mortgage discrimination, *D*, for census tract *j* with the following equation:

$${D}_{j}=\frac{\frac{{L}_{{minority},j}}{{L}_{j}}}{\frac{{H}_{{minority},j}}{{H}_{j}}},$$

where ${L}_{{minority},j}$ and ${L}_{j}$ representing the total number of mortgage loans to racial and ethnic minority and all individuals, respectively, in a given census tract in the years 1990–2000, and ${H}_{{minority},j}$ and ${H}_{j}$ representing the total number of households for racial and ethnic minority and all individuals, respectively, in a given census tract in the year 2000. A value < 1 suggests mortgage discrimination against racial and ethnic minority individuals, as the proportion of loans given to racial and ethnic minority individuals is lower than the proportion racial and ethnic minority residents in a given census tract. All data was normalized to 2010 census tract boundaries using the Longitudinal Tract Database [32].

### Statistical analysis

All analyses were conducted in R v4.2.1. Code to reproduce the study analyses can be accessed in the following GitHub repository: https://github.com/abasshkembi/noise-oej. We assessed the variability in the census tract workplace- and transportation-related noise pollution estimates as well as aggregated the estimates to various geographic scales (e.g., county, nationwide). We visualized spatial heterogeneity in noise with various choropleth maps. We characterized potential inequities to both sources of noise using exposure-risk ratios (ERR) [33]. The ERRs describe whether a specific racial and ethnic group, *j*, had disproportionately higher exposure to workplace and transportation noise relative to the nationwide prevalence using the following equation:

$${{ERR}}_{j}=\frac{\frac{{\sum }_{i=1}^{n}{\omega }_{i}{p}_{{ij}}}{{\sum }_{i=1}^{n}{p}_{{ij}}}}{\frac{{\sum }_{i=1}^{n}{\omega }_{i}{t}_{{ij}}}{{\sum }_{i=1}^{n}{t}_{{ij}}}}.$$

where ${\omega }_{i}$ is the prevalence of exposure (to either workplace or transportation noise) in census tract *i* across *n* total census tract; ${p}_{{ij}}$ is the population of racial and ethnic group *j* in census tract *i*; and ${t}_{{ij}}$ is the total population in census tract *i*. To estimate the ${{ERR}}_{j}$ for cumulative noise pollution, we replaced ${\omega }_{i}$ with the multiplication of the prevalence of workplace and transportation noise together. An ERR > 1 suggests that racial and ethnic group *j* was over-represented among the exposed population, compared to their nationwide representation. We estimated confidence intervals (CIs) for these RRs using 100 bootstrap samples. We used the 50^th^ percentile as the central estimate, and the 2.5^th^ and 97.5^th^ percentiles of the bootstrap sample for the 95% CIs.

#### Racial and ethnic composition and noise pollution

Using three generalized linear models, we estimated associations between the percentage of racial and ethnic minority individuals (as well as each subgroup) as the main independent variable and three outcomes of noise pollution as the main dependent variables: (i) cumulative high workplace and transportation noise using logistic regression; (ii) the prevalence of workplace noise pollution using Poisson regression; and (iii) the prevalence of transportation noise pollution using Poisson regression. Models (ii) and (iii) included an offset term for the total number of workers and total number of residents, respectively, as an offset to account for tract-to-tract differences in population.

We ran each of these three large model groups two separate times (for a total of six separate models), one for racial and ethnic minority individuals together, and one with independent covariates of racial and ethnic subgroups (non-Hispanic Black, Hispanic, non-Hispanic Asian, non-Hispanic American Indian/Alaskan Native, and non-Hispanic Native Hawaiian/Pacific Islander). We did not present independent associations for the proportion of non-Hispanic American Indian/Alaskan Native and Native Hawaiian/Pacific Islander residents in the main text because the lower population bounds of the fifth quintile were around 1%. These results are displayed in Appendix C of the Supplemental Materials. We classified the proportion of each racial and ethnic group into metropolitan/nonmetropolitan area (as defined by the Bureau of Labor Statistics in 2019)-specific quintiles, rather than nationwide quintiles, to account for city-to-city differences in racial and ethnic concentration. The effect estimates are presented relative to the first quintile. Each of six models adjusted for the census tract-level percentage of unemployed residents, urbanicity, population density in 2017 (natural spline with 8 degrees of freedom), and each tract centroid’s latitude/longitude (tensor product with 20 degrees of freedom) to account for spatial dependence of observations at a subregional level. Models displayed acceptable collinearity (for linear terms) and concurvity (for smoothed terms).

We conducted an additional sensitivity analysis to examine whether racial and ethnic inequities are still observed after accounting for two measures of SES. We further adjusted each model with the census tract-level percentage of low-income individuals and individuals without a high school diploma. These two measures of SES were not incorporated into our main model because SES could be a confounder but also is likely a mediator of the relationship between racial and ethnic composition and noise exposure. Since the central objective of this analysis is to assess the totality of racial and ethnic noise inequities, not racial and ethnic noise inequities independent of SES, our interest lies in the “total effects” of the racial and ethnic inequities (i.e., SES indicators not included) instead of “direct effects” (i.e., SES indicators are included).

#### Historical redlining, mortgage discrimination, and noise pollution

Using a linear model, we estimated the association between the 1930s historical redlining score as the main independent variable and the average prevalence of workplace- and transportation-related noise, stratified by 1990s mortgage discrimination. We used the average prevalence of workplace and transportation noise pollution to reflect cumulative noise pollution, rather than our previous percentile approach, as our main outcome because this analysis is restricted to a subset of census tracts within HOLC-defined neighborhoods (*n* = 12,823, or 18% of US census tracts) rather than the entire US. The model adjusted for census tract-level percentage of unemployed residents, population density in 2017 (natural spline with 8 degrees of freedom), and each tract centroid’s latitude/longitude (tensor product with 20 degrees of freedom) to account for spatial dependence of observations.

## Results

### Estimates of transportation- and work-related noise pollution

Aggregating noise prevalence estimates from 72,837 census tracts up to the nationwide level, an estimated 46.5 million (95% CI: 44.2–48.7) Americans were exposed to transportation-related noise pollution daily (>55 dBA over 24 h), equivalent to 14.3% (13.6–15.0) of the 324.6 million Americans in 2017. Among 150.6 million working Americans in 2017, an estimated 21.8 million (20.0–23.7) were exposed to workplace-related noise pollution daily (>85 dBA over 8 h), equivalent to 14.5% (13.3–15.8) of the working population or 6.7% (6.2–7.3) of the entire population.

**Fig. 2:**
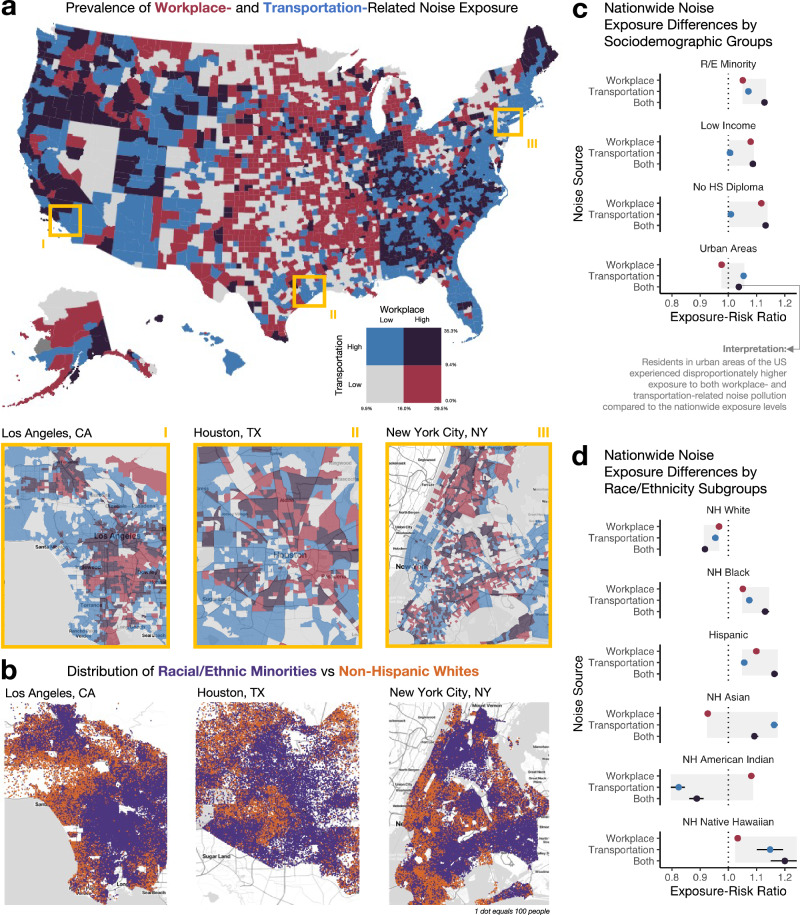
Workplace- and transportation-related noise pollution and sociodemographic inequities in the US. **a** Bivariate spatial distribution of the estimated prevalence of workplace (in red) and transportation (in blue) noise pollution at the county-level across the US, where counties in purple reflect those burdened by both. Census tract-level estimates are displayed for three large US cities (Los Angeles, California; Houston, Texas; and New York City, New York). **b** A dot-density map of racial and ethnic minority individuals (those not non-Hispanic White, in purple) vs non-Hispanic White individuals (in orange), where 1 dot represents 100 people. **c** Exposure-risk ratios and 95% CI by four sociodemographic factors for the prevalence of workplace noise, transportation noise, and high exposure to both. **d** Exposure-risk ratios and 95% CI by race and ethnicity groups for the prevalence of workplace noise, transportation noise, and exposure to both.

A bivariate, county-level map of the prevalence of workplace and transportation noise exposure is presented in Fig. 2a to summarize the census tract-level estimates, with counties facing high exposure to both sources of noise pollution shown in dark purple. The percentage of Americans exposed to transportation-related noise ranged from 0–100% among census tracts, with 75% of the estimates falling between 5.7 and 19.9%. The percentage of working Americans exposed to workplace-related noise by census tract ranged from 0 to 60%, with 75% of the estimates falling between 12.6 and 16.8%. Example census tract-level estimates are displayed for three US cities (to show regional differences across the US) below the county-level map, with respective racial dot-density maps shown in Fig. 2b. Generally, census tracts in Los Angeles, CA, Houston, TX, and New York City, NY with cumulatively high workplace and transportation noise pollution overlapped with areas densely populated with racial and ethnic minority individuals. Maps of all census tracts by workplace and transportation noise are displayed in Supplementary Figs. S1, S2.

In terms of relative exposure to both workplace and transportation noise pollution, neighborhoods with a higher proportion of racial and ethnic minority individuals faced disproportionately high cumulative exposure compared to nationwide levels (exposure-risk ratio [ERR] of 1.13, 95% CI: 1.12–1.13) (Fig. 2c and Supplementary Table S1). Similar cumulative inequities were observed among census tracts with more low-income individuals, those without a high school diploma, and in urban areas (ERRs ranged from 1.04 to 1.13). Specifically, by racial and ethnic subgroups, non-Hispanic (NH) Native Hawaiian, Hispanic, NH Black, and NH Asian communities had disproportionately higher cumulative exposure (NH Native Hawaiian ERR = 1.20, 95% CI: 1.15–1.25; Hispanic ERR = 1.16, 95% CI: 1.15–1.18; NH Black ERR = 1.13, 95% CI: 1.12–1.14; NH Asian ERR = 1.09, 95% CI: 1.08–1.11) (Fig. 2d and Supplementary Table S1).

### Associations between racial and ethnic composition and noise pollution

**Fig. 3:**
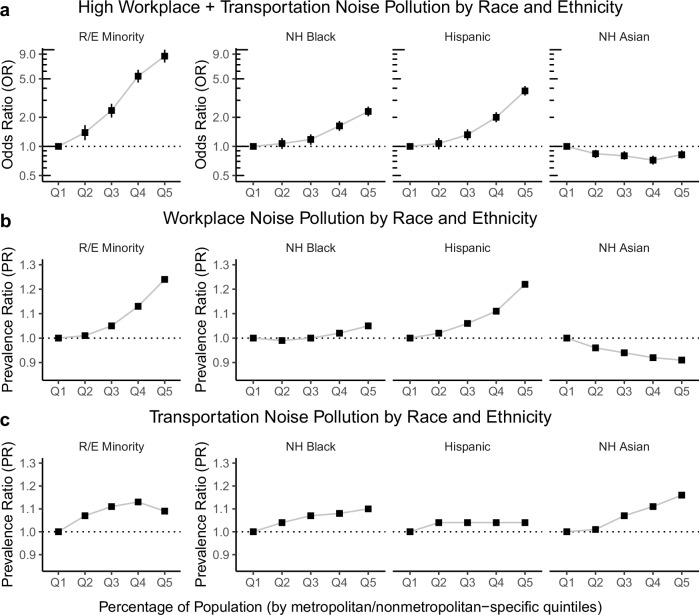
Associations between racial and ethnic composition and noise pollution. The relationship between census tract-level racial and ethnic (R/E) composition and (**a**) cumulatively high workplace and transportation noise, (**b**) workplace noise alone, and (**c**) transportation noise alone. A census tract was considered to have cumulatively high workplace and transportation noise pollution if was among the >75th percentile of both workplace and transportation noise pollution across the US. This relationship was modeled using logistic regression. Meanwhile, the relationships in (**b**) and (**c**) were modeled using Poisson regression with census tract-level number of people exposed as the outcome and the total number of people as an offset. All models accounted for urbanicity, the proportion of unemployed individuals, population density (natural spline with 8 degrees of freedom) and census tract centroid latitude/longitude (tensor product with 20 degrees of freedom). Note: NH, non-Hispanic. Quintiles for racial and ethnic subgroups were calculated using metropolitan/nonmetropolitan area cutoffs.

To identify nationwide racial and ethnic inequities in noise pollution, our first model evaluated the odds of a census tract being among the >75^th^ percentile of both transportation (>19.9% of residents exposed) and workplace (>16.8% of workers exposed) noise exposure prevalence (*n* = 3815, or 5.2%, of census tracts) per metropolitan/nonmetropolitan-specific quintile (Q) increase in the percentage of racial and ethnic minority individuals (Fig. 3a), reflecting racial and ethnic inequities to cumulatively high transportation and workplace noise pollution. After controlling for urbanicity, population density, unemployment, and location, we observed that census tracts with highest proportion of racial and ethnic minority individuals in a metropolitan area (Q5) had 8.59 (95% CI: 7.38–10.0) times higher odds of having both high transportation and workplace noise pollution compared to census tracts with lowest proportion of minority individuals (Q1). The largest cumulative inequities were observed among census tracts with high concentrations of Hispanic (OR = 3.75, 95% CI: 3.36–4.20 between Q5 vs Q1) and non-Hispanic Black (OR = 2.30, 95% CI: 2.05–2.58 between Q5 vs Q1) Americans. Census tracts with a higher percentage of non-Hispanic Asian Americans were significantly less likely of having cumulative high noise pollution.

To examine inequities to workplace or transportation noise pollution separately, we then evaluated the prevalence ratio of workplace noise pollution per quintile increase in the percentage of racial and ethnic minority individuals (Fig. 3b). After controlling for urbanicity, population density and unemployment, we observed that census tracts with highest proportion of racial and ethnic minority individuals in a metropolitan area (Q5) had 23.8% (95% CI: 23.6–24.0) higher percentage of workers exposed to workplace noise pollution compared to census tracts with the lowest proportion of racial and ethnic minority individuals (Q1). Similar to cumulative inequities, the largest inequity to workplace noise pollution were observed among larger percentages of Hispanic Americans (PR = 1.22, 95% CI: 1.21–1.22 between Q5 vs Q1) and non-Hispanic Black Americans (PR = 1.05, 95% CI: 1.05–1.05). On the other hand, census tracts with a higher percentage of non-Hispanic Asian had significantly smaller prevalence of workplace noise pollution. Inequities to transportation noise pollution among racial and ethnic minority individuals were less profound than to workplace noise pollution (PR of 1.09 vs 1.24, respectively, between Q5 and Q1 of racial and ethnic minority individuals) (Fig. 3c). Inequities to transportation noise were similar to those observed for workplace noise for the racial and ethnic subgroups, except among non-Hispanic Asian Americans (PR = 1.16, 95% CI: 1.16–1.16), who displayed the largest transportation-related inequity despite having a negative association with workplace noise. Detailed model results are presented in Supplementary Tables S2–S4.

We evaluated the robustness of our findings of racial and ethnic noise pollution inequities to potential confounding by the census tract-level percentage of low-income individuals and those without a high school diploma. The results of the sensitivity analysis remained consistent with our main study findings (Supplementary Fig. S4), although there were some differences with our main study results. For the odds of cumulatively higher workplace and transportation noise pollution, after also accounting for the two measures of socioeconomic status, census tracts with the highest proportion of non-Hispanic Asian Americans (Q5) had 1.10 (95% CI: 0.99–1.22) times higher odds of a cumulative inequity compared to census tracts with the lowest proportion of non-Hispanic Asians (Q1), despite a negative association being observed when not accounting for socioeconomic status (Fig. 3a). Otherwise, a general shrinking of effect sizes was observed across all racial and ethnic subgroups.

### Structural racism and cumulative noise inequities

**Fig. 4:**
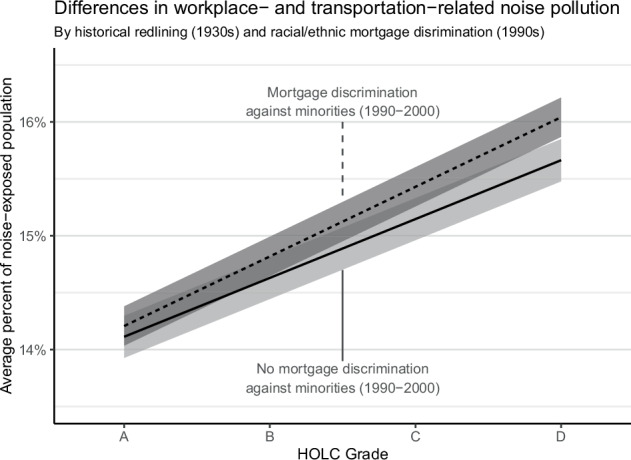
The census tract-level relationship between 1930s historical redlining and the average percent of workplace- and transportation-exposed individuals, stratified by census tract-level mortgage discrimination in the 1990s. The relationship was modeled using linear regrerssion accounting for the proportion of unemployed individuals, population density (natural spline with 8 degrees of freedom) and census tract centroid latitude/longitude (tensor product with 20 degrees of freedom).

To assess whether aspects of structural racism contributed to racial and ethnic inequities to noise, we investigated the relationship of the census tract-level historical redlining score, HRS (scale of 1–4, with 4 indicating the highest degree of historical redlining in the 1930s), and their associations with the average prevalence of workplace- and transportation-noise pollution (rather than the odds of >75^th^ percentile of workplace and transportation noise). This was stratified by mortgage discrimination against racial and ethnic minority individuals in the 1990s, otherwise referred to as “sustained discrimination.” HRS values are available for *n* = 12,823, or 18%, of all US census tracts, equivalent to nearly 46 million Americans, of which 23 million were working. This is because only urban areas with >40k people in the 1930s and 1940s were assessed by the HOLC, which equates to a subset of the US population today. After accounting for unemployment and population density, a change from 1 to 4 in the HRS among census tracts with sustained discrimination in the 1990s was associated with a 1.83% (95% CI: 1.66–2.01) higher average prevalence of workplace- and transportation-related noise pollution. Among census tracts without sustained discrimination, a change from 1 to 4 in the HRS among census tracts was associated with a 1.55% (95% CI: 1.37–1.74) higher average prevalence (Fig. 4).

## Discussion

While environmental injustices of noise pollution have been previously characterized in a handful of studies, much less is known about the cumulative injustices to workplace and environmental noise pollution together. Our study provides new evidence that racial and ethnic composition is associated with cumulatively high exposure to workplace- and transportation-related noise nationwide, providing a new lens with which to examine the cumulative impacts of environmental injustices in the US. This cumulative injustice likely burdens communities with large proportions of Hispanic and non-Hispanic Black Americans the most, as well as non-Hispanic Asian Americans when accounting for income and educational attainment. Our findings suggest that early 20^th^ century structural drivers of residential segregation, coupled with continued mortgage discrimination, could explain why these cumulative injustices in noise pollution are observed today.

Our findings were consistent with previous studies documenting environmental noise inequities. Casey et al. found that anthropogenic noise levels (estimated by the US National Park Service) were substantially higher for Asian, Hispanic, and Black Americans, a finding corroborated by our study [2]. Another study by Collins & Grineski utilizing transportation noise levels from the US Department of Transportation, as we did in this study, also found similar racial and ethnic inequities, despite using a different approach to estimate transportation exposures [34].

These previous studies did not examine workplace noise and little is known about occupational inequities to noise for comparison with our workplace-specific findings. At the time of this study, there were no nationwide studies examining racial and ethnic differences in occupational noise except for one of our previous studies using similar methods to estimate census tract-level workplace noise [17]. We can, however, compare occupational noise inequities to the few studies that have investigated inequities to other workplace stressors that are correlates with noisy jobs. The COVID-19 pandemic in particular highlighted how racial and ethnic minority individuals were more likely to work in “essential” jobs, placing them at greater risk of infection [35, 36]. Another nationwide study observed racial and ethnic inequities, particularly among Hispanic workers, to multiple chemical exposures [37]. Consistent with these previous studies, our study found substantial occupational noise inequities in communities with increasingly more Hispanic and non-Hispanic Black workers. This provides further evidence for how racial and ethnic minority individuals continue to be occupationally segregated into more hazardous labor today.

We anticipated that racial and ethnic inequities would be more profound for environmental noise rather than occupational noise, as we expect that land use decisions that influenced the placement of transportation-related noise sources (e.g., highways) to be very spatially concentrated. However, we observed the opposite, finding that occupational inequities can be just as, if not more, profound than environmental inequities. Results showed that census tracts with the highest proportion of racial and ethnic minority individuals in a metropolitan area had 1.24 times higher percentage of workers exposed to workplace noise pollution compared to the lowest proportion, while a difference of 1.09 times was observed for transportation-related noise pollution. This highlights the need to consider the cumulative impact of stressors from both environmental and occupational sources rather than focusing on environmental sources only.

Cumulative impacts, which refers to the totality of exposures and their impact on human health, has traditionally resulted in studies and policies considering multiple, overlapping chemical and non-chemical stressors, typically only assessing environmental sources of exposure given the robust availability of such data [38]. Examination of multiple sources of exposure, such as incorporating workplace exposures, has been lacking because information on place-specific workplace exposures (e.g., at the census tract level) has been understudied. This study demonstrates a feasible method to address this gap in cumulative impact assessment by allowing various stakeholders to efficiently leverage existing estimates of chemical and non-chemical stressors at the census tract-level with place-based estimates of workplace exposures, in this case, noise.

The central issue of this approach, however, is that environmental and occupational regulations remain disjointed in the US. Thus, federal/state environmental and occupational agencies have limited power in controlling exposures occurring both in a workplace and in a community. We can look to the US EPA’s Toxic Substances and Control Act (TSCA) as a reasonable route for environmental regulations to consider workplace sources of exposures. In 2016, an amendment to TSCA gave the EPA authority to consider chemical exposures among workers when conducting environmental assessments.[39] Although noise pollution is not in the scope of TSCA, it demonstrates how reform to other existing environmental regulation, such as the EPA’s Noise Control Act of 1972, can allow the consideration of the cumulative impacts of noise pollution. As mentioned in the introduction, the Noise Control Act has not been enforced by the EPA since the Office of Noise Abatement and Control (ONAC) was defunded in 1981 [5]. In light of these circumstances and the findings of this study, we urge the EPA to not only reconsider refunding ONAC to regulate environmental noise pollution, but also for policymakers and legislators to consider similar amendments to the Noise Control Act as in TSCA which would allow the EPA to consider both environmental- and workplace-related noise in their assessments. For other governmental departments, such as the Department of Transportation, and local urban planners where ambient noise pollution is a focus but controlling workplace noise is outside of their scope, we recommend these agencies consider the spatial heterogeneity of workplace noise in their assessments of ambient noise to better target communities with high noise exposures environmentally and occupationally, rather than just high environmental exposures. For example, DOT and local urban planners could take into consideration communities with high workplace noise when developing new projects (e.g., a new highway), so that communities who do not have reprieve from noise at work are not doubly burdened by environmental noise when they return home.

While the regulation of noise pollution at the federal level continues to be resolved, our findings of cumulatively high workplace and transportation noise pollution across US urban centers demonstrates the role local city governments can play in controlling noise pollution and reducing racial and ethnic inequities. While the inequities observed between historically redlined and non-redlined neighborhoods appeared small (1.83% and 1.55% difference for neighborhoods with and without continued discrimination, respectively), such differences within populous urban centers still reflect hundreds of thousands of exposed individuals. Our findings suggest that particular attention should be given towards controlling noise pollution in neighborhoods historically marginalized with sustained mortgage discrimination to help reverse the environmental injustices caused by redlining. The British Medical Association recommends that reduction of traffic speeds in cities is the cheapest intervention with the largest reductions to transportation-related noise pollution [40]. Policies which reduce racial and ethnic mortgage discrimination could also improve workplace noise pollution by likely reducing income inequality and improving educational access in historically marginalized neighborhoods, which are known contributors to occupational health disparities [41].

### Limitations

Our analyses were conducted at an ecological level and our findings are susceptible to the ecological fallacy. Our analytical approach of two distinct sources of noise pollution did not allow us to examine cumulative workplace and environmental exposures at the individual level. Therefore, our approach cannot determine the degree of co-exposure to environmental and occupational noise, although our main assumption is that there is some overlap in those exposed to workplace and transportation noise. While we did not model the likelihood of co-exposure in this study, we can theoretically consider the implications of our findings under two extreme, possible scenarios with our analytical approach: (i) the “best case scenario,” in which individuals exposed to workplace noise could be an entirely distinct population from those exposed to transportation noise; and (ii) the “worst case scenario,” where individuals exposed to workplace noise completely overlap with those exposed to transportation noise. In the “best case scenario”, a large portion of the population exposed to noise in the workplace would be entirely overlooked by policies seeking to reduce the adverse effects of noise if only environmental sources of noise are assessed, and vice versa. These policies would help some people exposed, but likely ignore many more. In the “worst case scenario”, reducing transportation noise for the individuals cumulatively exposed may still leave individuals exposed to levels of noise hazardous to human health in their workplace, and vice versa. Such policies would help most people exposed, but not help those people enough. These two scenarios reinforce that, despite the limitations of our approach, the implications of our study findings remain relevant to incorporating occupational exposures into cumulative impact assessments.

Another limitation of this study is the grouping of the three main sources of transportation noise (i.e., road, rail, and air) into a single measure of transportation-related noise pollution prevalence. While this was intentional to create a balance in the occupational and transportation noise measures (otherwise we would have assessed one occupational measure compared to three transportation measures), this means that our study does not provide information about geographical variation to different types of transportation noise. Regardless, the main focus of this study was the assessment of occupational and environmental noise pollution together, rather than delving into different sources of environmental noise.

Our approach to estimate workplace noise cannot disentangle the contributions of ambient noise exposure during work for outdoor workers. For example, a construction worker may be exposed to industrially-produced sources of noise (e.g., using a hammer at a construction site) as well as ambient sources of noise (e.g., an airplane from a nearby airport flies over the construction site). While both sources of noise are theoretically captured in the noise job exposure matrix, we cannot discern (1) the total contribution of industrially-produced vs ambient sources of noise, nor (2) the local contributions of a particular road, railway, or airport to workplace noise since the American Community Survey does not indicate whether the home census tract of a worker aligns with the census tract where they work. Despite these limitations, our approach to estimate workplace noise likely incorporates both industrially-produced and ambient sources of noise.

We likely undercounted the number of individuals exposed to transportation-related noise, particularly airplane noise. The WHO recommends reducing 24-h road noise to 53 dBA, rail noise to 54 dBA, and airplane noise to 45 dBA to sufficiently protect against the noise-related adverse impacts of each transportation type [3]. With the tradeoff of undercounting exposed individuals, we utilized a conservative threshold of 24-hr L_Aeq_ noise levels above 55 dBA to determine transportation noise exposure for the benefit of better identifying communities exposed to all transportation sources of noise rather than just one. Lastly, this study examined population-level noise exposure and not dose (i.e., the actual level of noise entering the human ear). Additionally, varying occupational behaviors, such as hearing protection device use during work, or community factors, such as housing quality, would affect the actual dose. Not accounting for these factors likely underestimates the magnitude of the cumulative inequities.

## Conclusions

Current environmental and occupational regulations in the US do not adequately protect racial and ethnic minority communities from noise pollution. Ignoring sources of noise from the workplace likely under-counts noise-related environmental injustices. Communities marginalized by historical housing discrimination and sustained mortgage discrimination should be targeted to reduce cumulatively high transportation- and workplace-related noise pollution in the US.

## Supplementary information

Supplementary Material

## Data Availability

All data used in this analysis are publicly available. The final, simulated workplace and transportation noise prevalences by census tract are available at https://github.com/abasshkembi/noise-oej. Census tract-level racial and ethnic composition are available from the ‘tigris‘ R package (https://cran.r-project.org/package=tigris). Census tract-level 1930s historical redlining data are available at 10.3886/E141121V2, and 1990s mortgage discrimination data are available at 10.3886/ICPSR39093.v1. Further documentation of the data sources and processed data is available in the following GitHub repository: https://github.com/abasshkembi/noise-oej.

## Code availability

Replication code and the full dataset required to reproduce the results are maintained in the following GitHub repository: https://github.com/abasshkembi/noise-oej.
