# Supplementary material for: Racial and ethnic inequities to noise pollution from transportation- and work-related sources in the United States
Source: J Expo Sci Environ Epidemiol. 2025 Jul 17;36(1):211–20. doi: 10.1038/s41370-025-00795-x (PMC12795750; doi:10.1038/s41370-025-00795-x)
Supplement: Supplementary file 1 — Supplementary Material [file 41370_2025_795_MOESM1_ESM.docx]

*Supplementary Material for*

**Racial and ethnic inequities to noise pollution from transportation- and work-related sources in the United States**

Abas Shkembi^1,*^; Keshav Patel^1^; Lauren M. Smith^1^; Helen C.S. Meier^2^; Richard L. Neitzel^1,*^

^1^Department of Environmental Health Sciences, University of Michigan School of Public Health, Ann Arbor, MI, USA

^2^Survey Research Center, University of Michigan, Ann Arbor, MI, USA

**^*^Correspondence**

Abas Shkembi, Department of Environmental Health Sciences, University of Michigan School of Public Health, 1415 Washington Heights, Ann Arbor, MI 48109. e: [ashkembi@umich.edu](mailto:ashkembi@umich.edu)

Richard L. Neitzel, Department of Environmental Health Sciences, University of Michigan School of Public Health, 1415 Washington Heights, Ann Arbor, MI 48109. e: [rneitzel@umich.edu](mailto:rneitzel@umich.edu)

­­­­­­­_____________________________________________________________________________________

**Table of Contents**

**Appendix A**. Creation of transportation noise prevalence by census tract

**Appendix B**. Creation of workplace noise prevalence by census tract

**Appendix C**. Supplemental Tables and Figures

**Appendix A**. Creation of transportation noise prevalence by census tract

**Objective**

To construct estimates for the prevalence of exposure to transportation-related noise pollution for each census tract across the US.

**Data Sources**

*Department of Transportation National Transportation Noise Map.* The 2018 U.S. Department of Transportation Bureau of Transportation Statistics National Transportation Noise Map presents 24-hr A-weighted sound levels (L_eq_) to represent average sound levels (dBA) in a uniform 30m grid for road and rail noise sources and a dynamic grid for air noise sources. Noise levels below 45 dBA were not estimated. Raster files were accessed at <https://www.bts.gov/geospatial/national-transportation-noise-map> on December 12, 2023 for each US state. Alaska, Hawaii, South Dakota, and Wyoming had no rail noise estimates.

*2010 Decennial Census*. We accessed population counts at the census block level from the 2010 Decennial Census using the `tidycensus` R package. Specifically, population counts were accessed from “P001001” from the Summary File 1 dataset.

**Data Processing**

For a given state, raster files for road, rail, and aviation noise levels were converted to points using the centroid of each grid. The centroid points were then assigned to their corresponding, overlapping census block. Each transportation noise estimate for a state was then compiled into a single dataset with noise levels, noise source (road, rail or air), each census blocks unique geographic identifier (GEOID), and associated geographic information. For computational efficiency of our simulation, we removed census blocks with no individuals living there.

**Simulation**

For each census block, we estimated the number of individuals potentially exposed to transportation noise (overexposure defined as >55 dBA for any of road, rail, or air sources) to be aggregated up to the census tract level. The simulations were done at the census block level and then aggregated to the census tract level, rather than performing the simulations at the census tract level, to reduce the potential of overestimating the number of individuals exposed because blocks are the smallest geographical unit of analysis (e.g., a city block bounded on all sides by streets) in the Census.

*Scenario 1: Census blocks with no noise measurements.* We assumed that all people living in a census block with zero noise samples are assumed to have no transportation-related noise exposure.

*Scenario 2: Census blocks with one noise measurement.* We assumed that all people living in a block with only one noise sample are all assumed to be exposed to that same, single noise level. Thus, if that single noise measurement was above 55 dBA, all individuals in the census block were considered overexposed; otherwise, no individuals were considered overexposed.

*Scenario 3: Census blocks with at least two noise measurements.* For each census block, we calculated the probability of overexposure ($P_{x,b}$) to transportation noise as the sum of all noise measurements >55 dBA ($N_{>55,b}$) divided by the total number of noise measurements ($N_{b}$) for a given census block *b*:

$$P_{x,b}=\frac{N_{>55,b}}{N_{b}}$$

All residents in census blocks with $P_{x,b}$=0 were not considered overexposed. For census blocks with a $P_{x,b}$> 0, we took a Monte Carlo simulation approach to estimate the percentage of residents overexposed. For each census block, we randomly sampling from a binomial distribution $\text{Bin}(n={Pop}_{g} , p=P_{x,b})$ to estimate the number of residents overexposed to transportation noise, where ${Pop}_{g}$ reflect the total number of residents in census block *g*. We repeated this simulation 100 times.

For a given simulation iteration, we aggregated the number of individuals overexposed simulations to the census tract-level to calculate the estimated percentage of residents exposed to transportation noise >55 dBA.. We estimated the mean, median, standard deviation, and 2.5^th^ percentile, and 97.5^th^ percentile for each tract across the 100 iterations. Estimates for census blocks with <20 residents were excluded.

**Results**

Census tract-level environmental noise prevalence ranged from 0 to 100%. The simulations were generally not skewed (**Figure A1**), with a Pearson’s correlation of the median and mean prevalence near 1 (r >0.99), although the simulations were more variable at the extreme (0% and 100%). As a result, we used median prevalences, rather than mean, as the final point estimate for a given census tract.

| 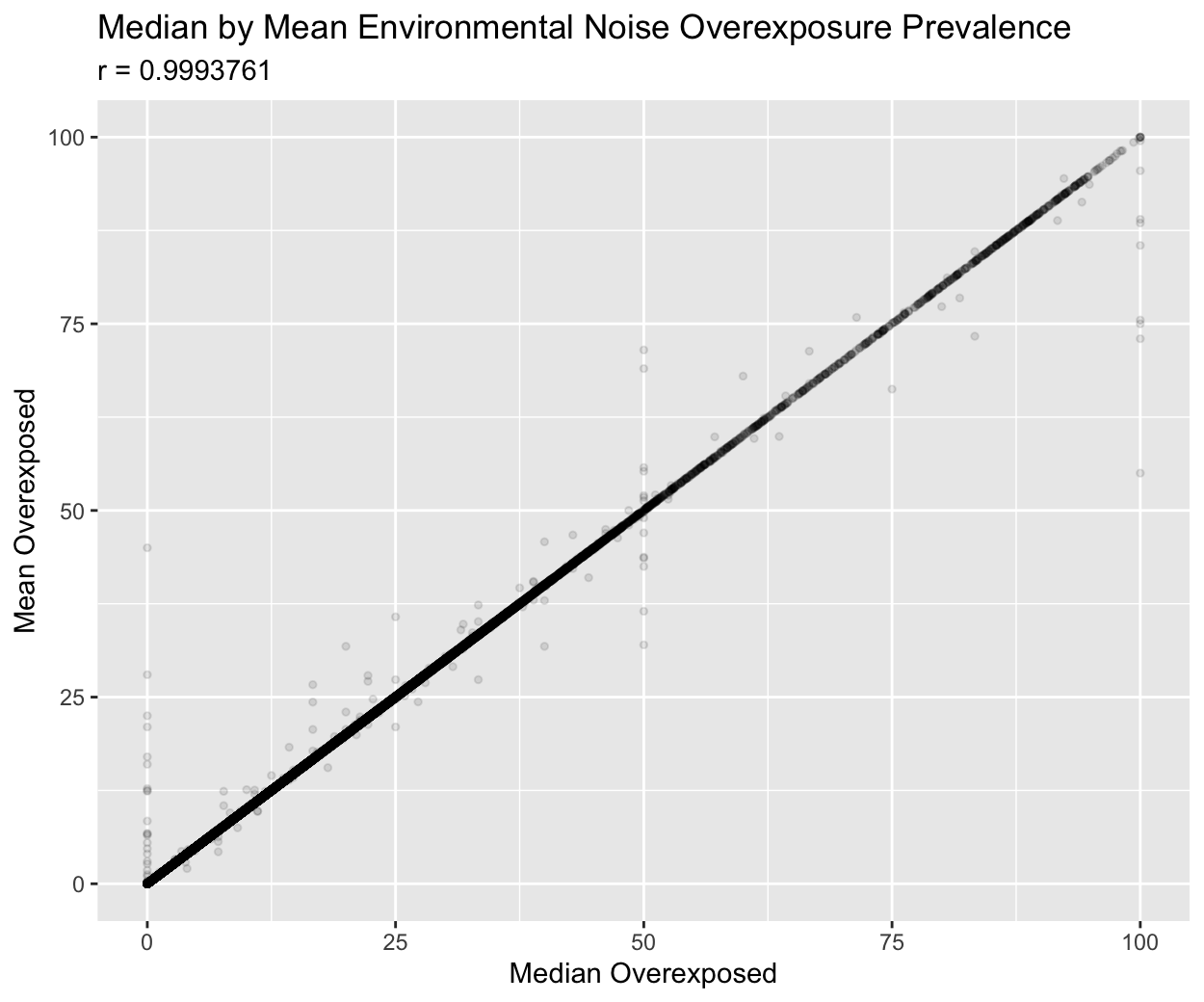 |
| --- |
| **Fig A1**. Census tract-level scatterplot of the median prevalence and mean prevalence (in percentage of total population) |

**Data and Code Availability**

Replication code and the input data required to reproduce the simulations are maintained in the following GitHub repository: <https://github.com/abasshkembi/noise-oej>.

**Appendix B**. Creation of workplace noise prevalence by census tract

**Objective**

To construct estimates for the prevalence of exposure to occupational-related noise pollution for each census tract across the US.

**Data Sources**

*NoiseJEM.* The Noise Job Exposure Matrix was constructed using noise exposure measurements from industries in the U.S and Canada from 1960-2015 (<https://noise.shinyapps.io/noiseJEM/>). Specifically, we utilized previously published posterior mean estimates and standard deviations of the 8-hr noise level for each major Standard Occupational Classification (SOC) code, as reported in Table 3 of Roberts et al. (2018).^[[1]](#footnote-1)^

*2015-2019 American Community Survey.* We accessed employment counts by major census occupational groups at the census tract level using the `tigris` R package. We utilized 5-year ACS from 2015 to 2019 to gather total employment count for each US census tract and breakdowns by major occupational groups for 2017. There are 22 major non-military groups. The ACS follows the Census Occupation Code structure for classifying occupational groups and can be linked to SOC structure utilized in the NoiseJEM.

**Simulations**

For each state, 10,000 Monte Carlo simulations were run at the census tract level to estimate the proportion of employed individuals overexposed to occupational noise. We used a random sampling method to simulate the noise exposure level for each employed person, assuming that the posterior mean and standard deviation estimates for each major occupational code represented a normal distribution. The simulated noise levels for each worker was then determined to be overexposed or not (defined as ≥85 dBA). Each simulation was aggregated to calculate the percentage of employed individuals overexposed to occupational noise in each tract. Fifty-seven tracts with less than 21 employed individuals were excluded from the aggregated dataset because the sample was not large enough. We calculated mean, median, standard deviation, 2.5% and 97.5% quantiles for percent overexposed per tract.

**Results**

Census tract-level workplace noise prevalence ranged from 0 to 60%. The simulations were generally not skewed (Figure B2), with a Pearson’s correlation of the median and mean prevalence near 1 (r >0.99, Figure B1). The simulations were more variable among census tracts with ≤50 employed residents (Figure B3). As a result, we used median prevalences, rather than mean, as the final point estimate for a given census tract.

| 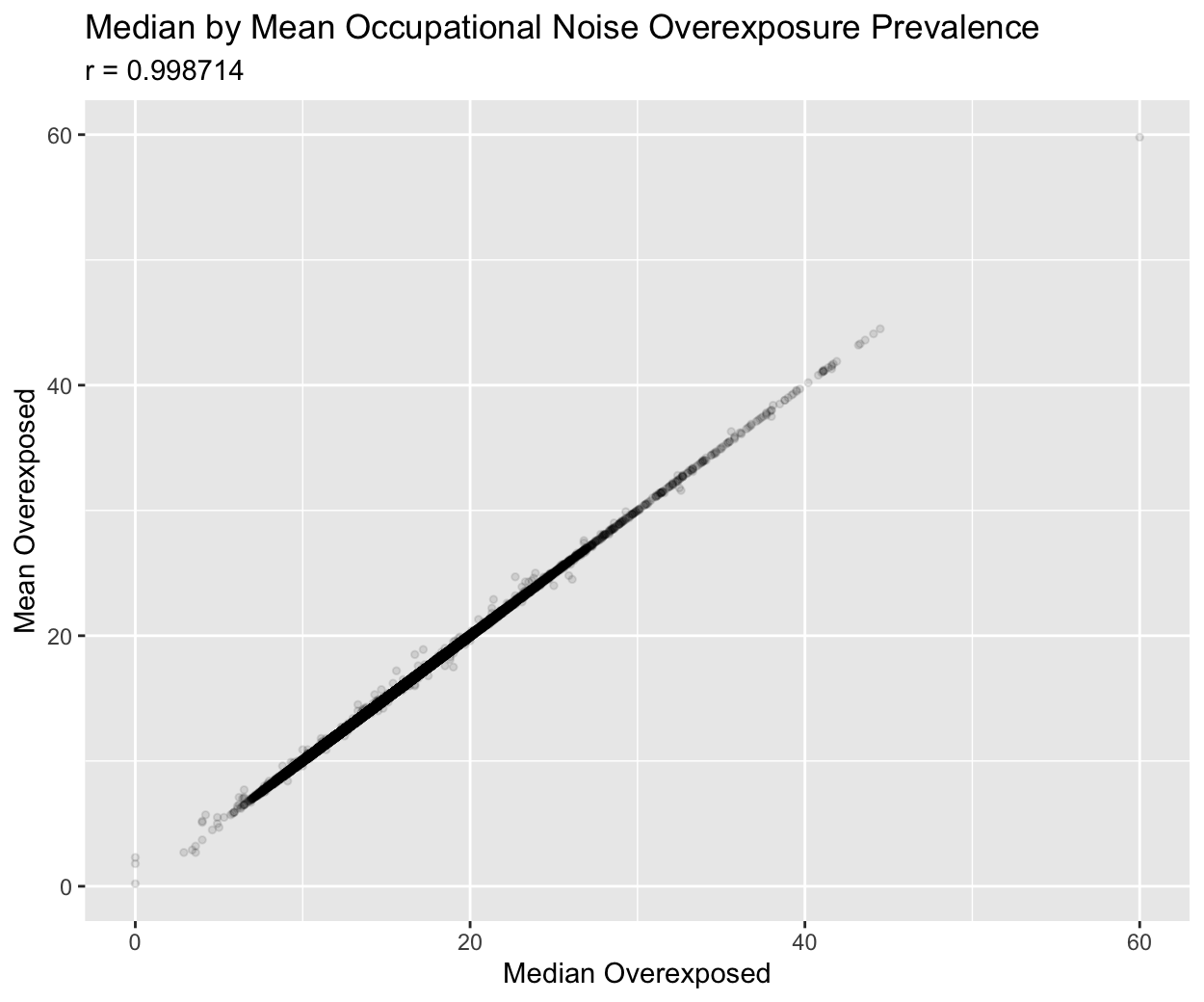 |
| --- |
| **Fig B1**. Census tract-level scatterplot of the median prevalence and mean prevalence (in percentage of total population) |

| 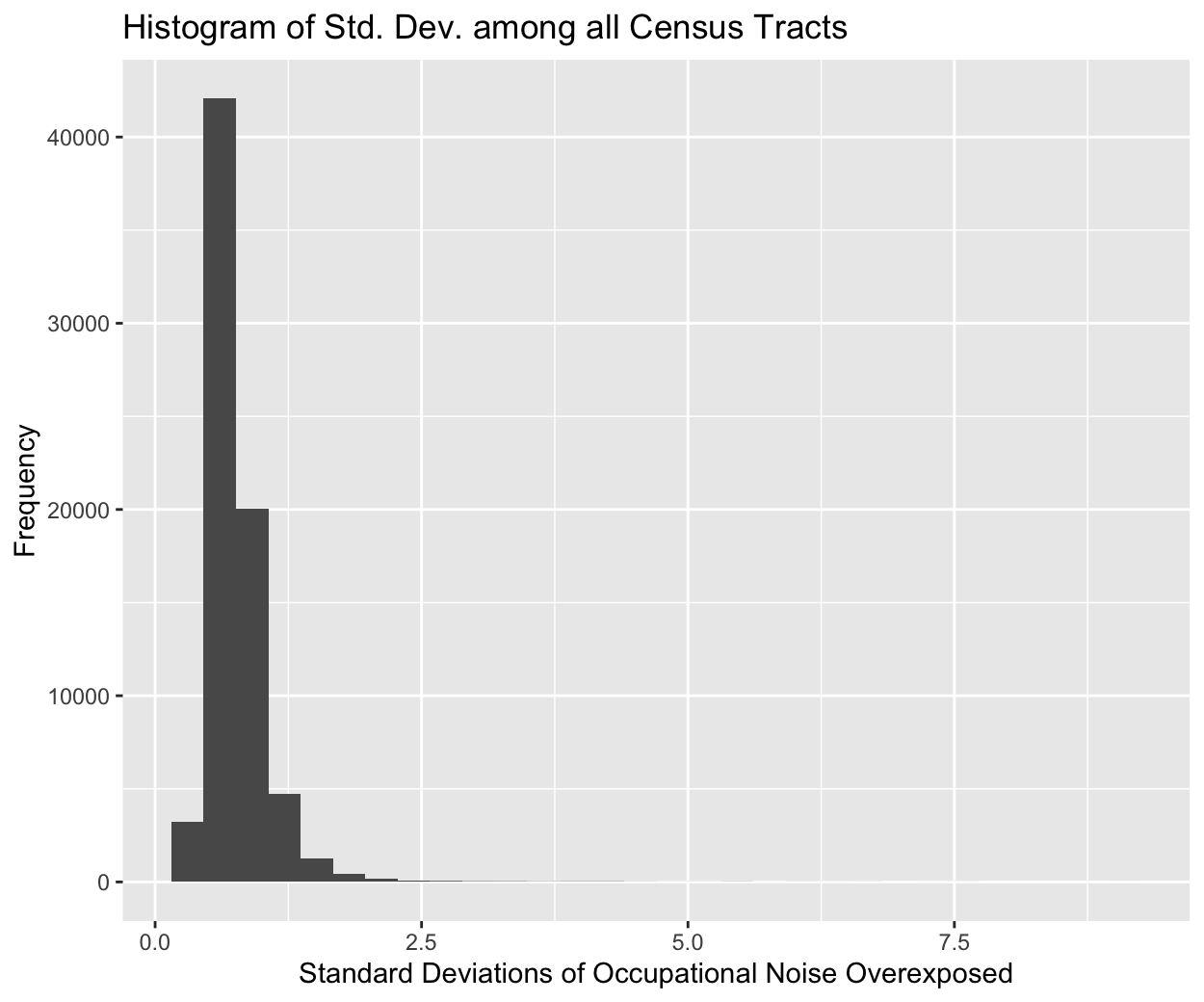 |
| --- |
| **Fig B2**. Census tract-level scatterplot of the median prevalence and mean prevalence (in percentage of total population) |

| 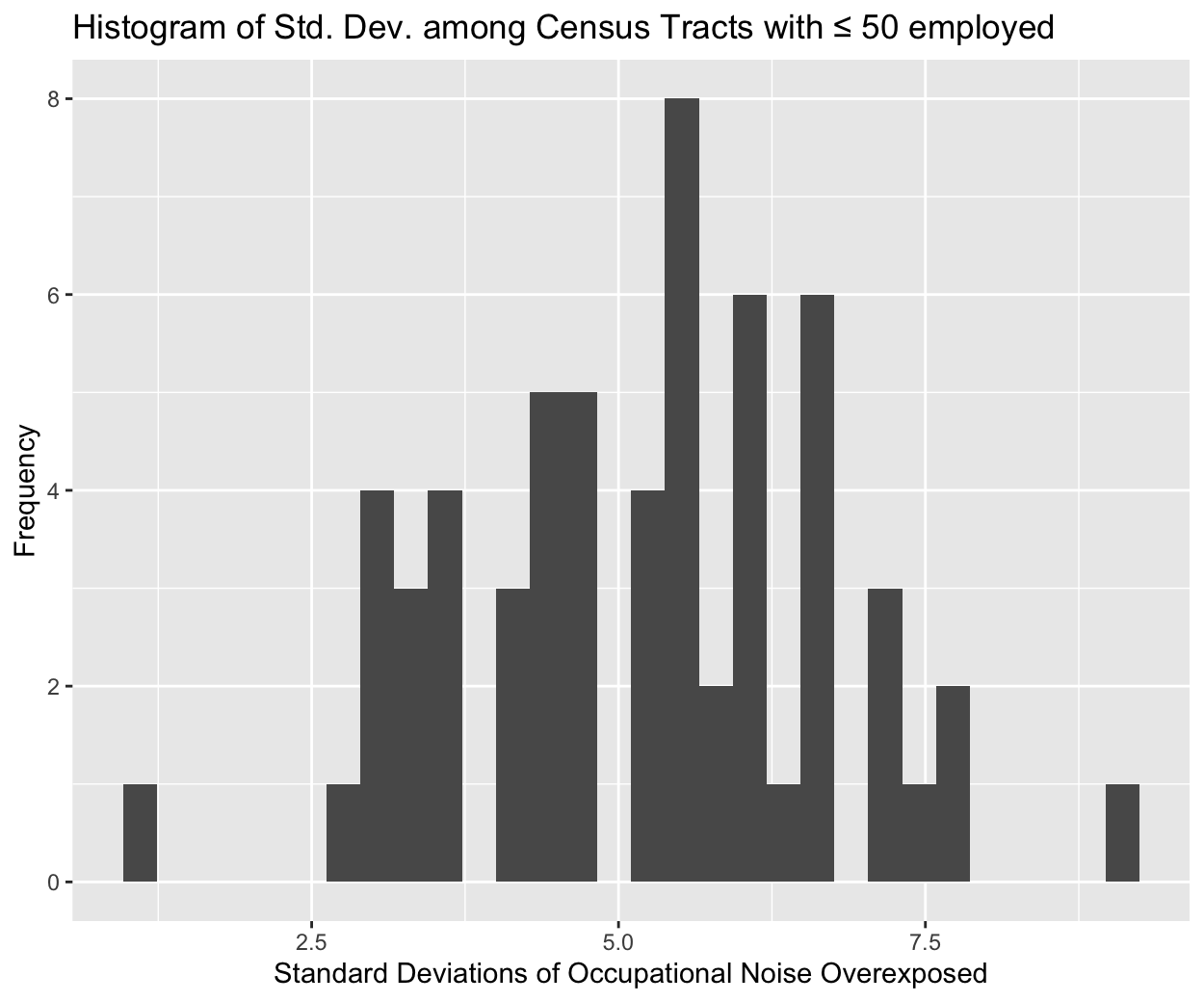 |
| --- |
| **Fig B3**. Census tract-level scatterplot of the median prevalence and mean prevalence (in percentage of total population) |

**Data and Code Availability**

Replication code and the input data required to reproduce the simulations are maintained in the following GitHub repository: <https://github.com/abasshkembi/noise-oej>.

**Appendix C**. Supplemental Tables and Figures

| **Table S1.** Exposure-risk ratios (ERRs) and 95% CI by sociodemographic factors for the prevalence of workplace noise, transportation noise, and high exposure to both. Estimates are reflected in Figure 2 in the main text. | | | |
| --- | --- | --- | --- |
| **Sociodemographic characteristic** | **Workplace** | **Transportation** | **Both** |
| Low-income | 1.08 (1.08, 1.08) | 1.01 (1.00, 1.01) | 1.09 (1.08, 1.09) |
| No high school diploma | 1.12 (1.12, 1.12) | 1.01 (1.00, 1.05) | 1.13 (1.12, 1.14) |
| Urban areas | 0.98 (0.98, 0.98) | 1.06 (1.05, 1.06) | 1.04 (1.03, 1.04) |
| Racial and ethnic minority | 1.05 (1.05, 1.05) | 1.07 (1.07, 1.08) | 1.13 (1.12, 1.13) |
| *White (Non-Hispanic)* | 0.97 (0.97, 0.97) | 0.95 (0.95, 0.96) | 0.92 (0.91, 0.92) |
| *Black (Non-Hispanic)* | 1.05 (1.05, 1.05) | 1.08 (1.06, 1.08) | 1.13 (1.12, 1.14) |
| *Hispanic* | 1.10 (1.10, 1.10) | 1.06 (1.05, 1.06) | 1.16 (1.15, 1.17) |
| *Asian (Non-Hispanic)* | 0.93 (0.92, 0.93) | 1.16 (1.15, 1.17) | 1.09 (1.08, 1.10) |
| *American Indian/Alaskan Native (Non-Hispanic)* | 1.08 (1.07, 1.09) | 0.82 (0.80, 0.84) | 0.88 (0.86, 0.91) |
| *Native Hawaiian/Pacific Islander (Non-Hispanic)* | 1.03 (1.03, 1.04) | 1.15 (1.10, 1.18) | 1.20 (1.15, 1.25) |

| **Table S2.** The relationship between census tract-level racial and ethnic (R/E) composition and cumulatively high workplace and transportation noise, as shown in Figure 3a. A census tract was considered to have cumulatively high workplace and transportation noise pollution if was in the 75th percentile of both workplace and transportation noise pollution across the US. This relationship was modeled using logistic regression. All models accounted for urbanicity, the proportion of unemployed individuals, population density (natural spline with 8 degrees of freedom) and census tract centroid latitude/longitude (tensor product with 20 degrees of freedom). Note: M/NM, metropolitan/nonmetropolitan; NH, non-Hispanic; AIAN, American Indian and Alaskan Native; NHPI, Native Hawaiian and Pacific Islander. | | | | | | |
| --- | --- | --- | --- | --- | --- | --- |
| **M/NM Quintile** | **R/E minority^1^** | **NH Black^2^** | **Hispanic^2^** | **NH Asian^2^** | **NH AIAN^2^** | **NH NHPI^2^** |
| Q1 | 1 (ref) | 1 (ref) | 1 (ref) | 1 (ref) | 1 (ref) | 1 (ref) |
| Q2 | 1.39 (1.16, 1.66) | 1.07 (0.94, 1.22) | 1.07 (0.93, 1.22) | 0.84 (0.76, 0.92) | 1.08 (0.97, 1.21) | 0.90 (0.79, 1.01) |
| Q3 | 2.35 (1.99, 2.76) | 1.18 (1.04, 1.33) | 1.32 (1.16, 1.50) | 0.80 (0.73, 0.89) | 0.98 (0.86, 1.11) | 0.85 (0.75, 0.97) |
| Q4 | 5.33 (4.58, 6.20) | 1.63 (1.45, 1.83) | 2.00 (1.78, 2.26) | 0.72 (0.65, 0.80) | 0.96 (0.86, 1.07) | 0.79 (0.69, 0.92) |
| Q5 | 8.59 (7.38, 10.0) | 2.30 (2.05, 2.58) | 3.75 (3.36, 4.20) | 0.82 (0.75, 0.91) | 1.18 (1.07, 1.31) | 0.91 (0.82, 1.01) |

| **Table S3.** The relationship between census tract-level racial and ethnic (R/E) composition and workplace noise, as shown in Figure 3b. The relationships were modeled using Poisson regression with census tract-level number of people exposed as the outcome and the total number of people as an offset. All models accounted for urbanicity, the proportion of unemployed individuals, population density (natural spline with 8 degrees of freedom) and census tract centroid latitude/longitude (tensor product with 20 degrees of freedom). Note: M/NM, metropolitan/nonmetropolitan; NH, non-Hispanic; AIAN, American Indian and Alaskan Native; NHPI, Native Hawaiian and Pacific Islander. | | | | | | |
| --- | --- | --- | --- | --- | --- | --- |
| **M/NM Quintile** | **R/E minority** | **NH Black** | **Hispanic** | **NH Asian** | **NH AIAN** | **NH NHPI** |
| Q1 | 1 (ref) | 1 (ref) | 1 (ref) | 1 (ref) | 1 (ref) | 1 (ref) |
| Q2 | 1.01 (1.01, 1.01) | 0.99 (0.99, 0.99) | 1.02 (1.02, 1.02) | 0.96 (0.96, 0.97) | 0.96 (0.96, 0.96) | 0.98 (0.98, 0.98) |
| Q3 | 1.05 (1.05, 1.05) | 1.00 (1.00, 1.00) | 1.06 (1.06, 1.06) | 0.94 (0.93, 0.94) | 0.99 (0.98, 0.99) | 1.00 (0.99, 1.00) |
| Q4 | 1.13 (1.12, 1.13) | 1.02 (1.01, 1.02) | 1.11 (1.10, 1.11) | 0.92 (0.92, 0.92) | 1.00 (1.00, 1.00) | 0.99 (0.99, 1.00) |
| Q5 | 1.24 (1.24, 1.24) | 1.05 (1.05, 1.05) | 1.22 (1.21, 1.22) | 0.91 (0.90, 0.91) | 0.99 (0.99, 0.99) | 1.01 (1.01, 1.01) |

| **Table S4.** The relationship between census tract-level racial and ethnic (R/E) composition and transportation noise, as shown in Figure 3c. The relationships were modeled using Poisson regression with census tract-level number of people exposed as the outcome and the total number of people as an offset. All models accounted for urbanicity, the proportion of unemployed individuals, population density (natural spline with 8 degrees of freedom) and census tract centroid latitude/longitude (tensor product with 20 degrees of freedom). Note: M/NM, metropolitan/nonmetropolitan; NH, non-Hispanic; AIAN, American Indian and Alaskan Native; NHPI, Native Hawaiian and Pacific Islander. | | | | | | |
| --- | --- | --- | --- | --- | --- | --- |
| **M/NM Quintile** | **R/E minority** | **NH Black** | **Hispanic** | **NH Asian** | **NH AIAN** | **NH NHPI** |
| Q1 | 1 (ref) | 1 (ref) | 1 (ref) | 1 (ref) | 1 (ref) | 1 (ref) |
| Q2 | 1.07 (1.07, 1.07) | 1.04 (1.04, 1.05) | 1.04 (1.04, 1.04) | 1.01 (1.01, 1.02) | 1.08 (1.08, 1.08) | 0.99 (0.99, 0.99) |
| Q3 | 1.11 (1.11, 1.11) | 1.07 (1.06, 1.07) | 1.04 (1.04, 1.04) | 1.07 (1.01, 1.02) | 1.02 (1.02, 1.02) | 0.94 (0.94, 0.94) |
| Q4 | 1.13 (1.13, 1.13) | 1.08 (1.08, 1.08) | 1.04 (1.04, 1.04) | 1.11 (1.11, 1.11) | 1.03 (1.02, 1.03) | 0.97 (0.97, 0.97) |
| Q5 | 1.09 (1.08, 1.09) | 1.10 (1.10, 1.10) | 1.04 (1.04, 1.05) | 1.16 (1.16, 1.16) | 1.04 (1.04, 1.04) | 0.96 (0.96, 0.97) |

| 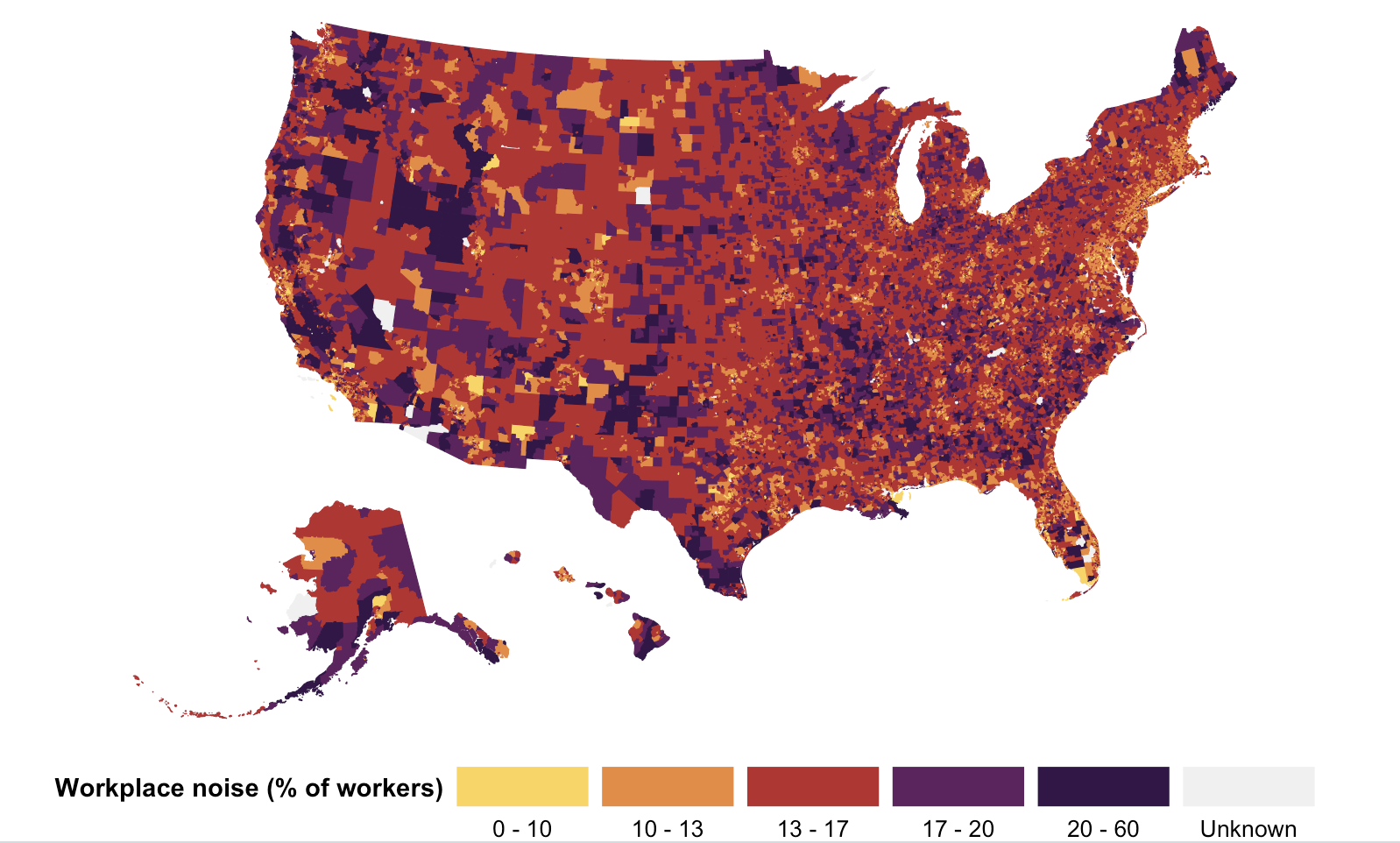 |
| --- |
| **Figure S1**. Prevalence of work-related noise exposure across US census tracts with working population >20 workers. The map displays the 0–10th percentile, 10–25th percentile, 25–75th percentile, 75–90th percentile, and 90–99th percentile. |

| 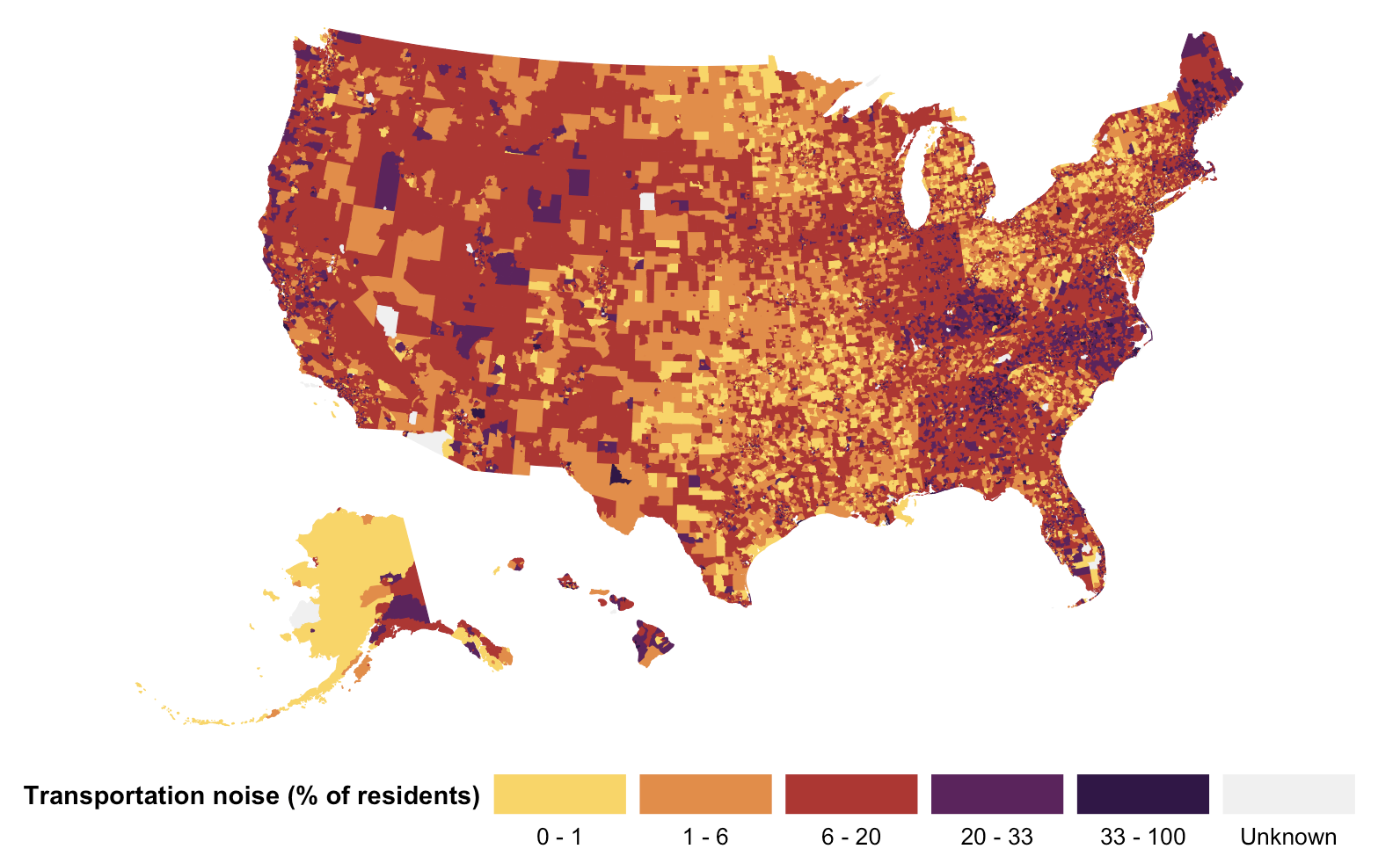 |
| --- |
| **Figure S2**. Prevalence of transportation-related noise exposure across US census tracts with total population >20 people. The map displays the 0–10th percentile, 10–25th percentile, 25–75th percentile, 75–90th percentile, and 90–99th percentile. |

| 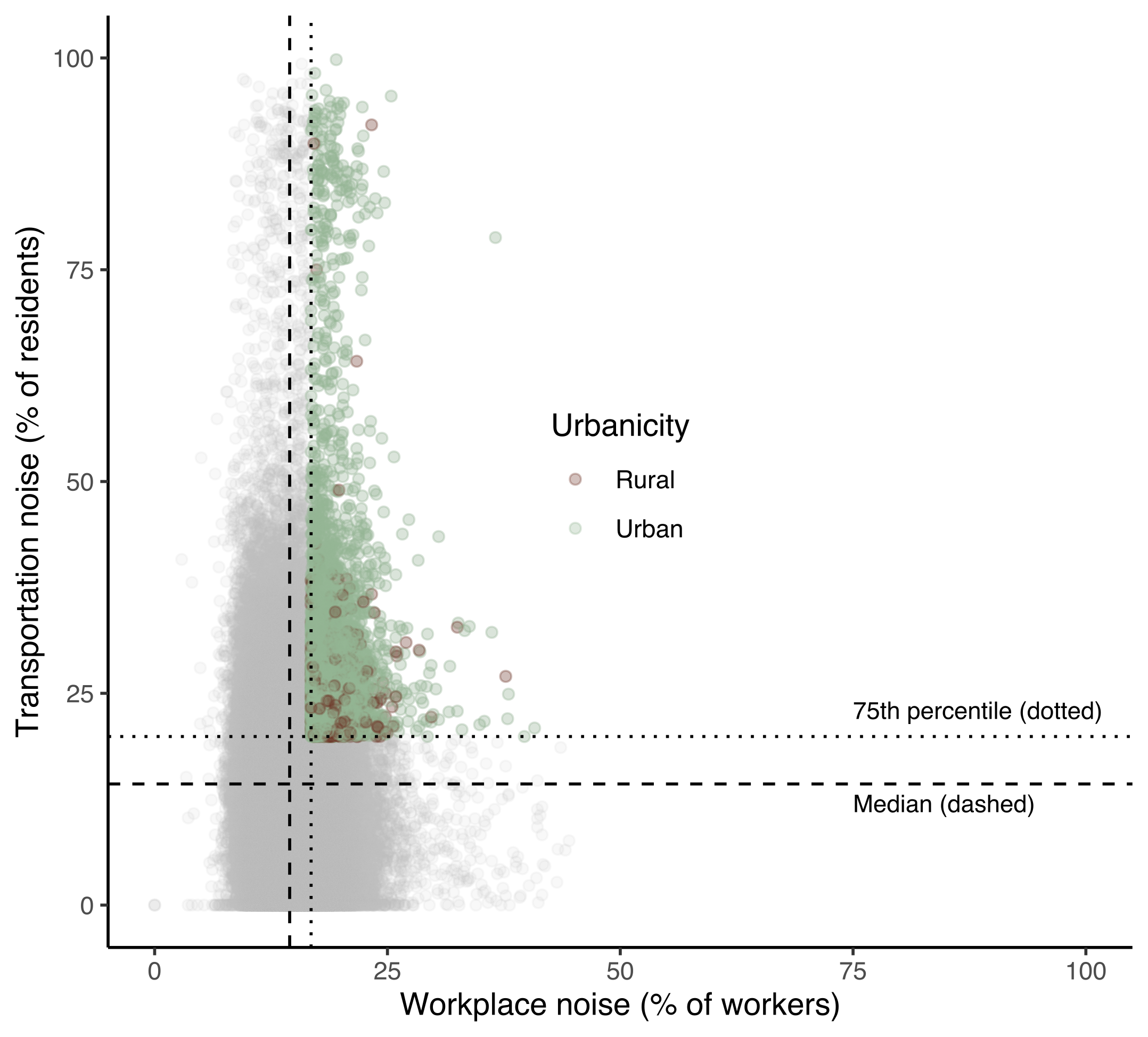 |
| --- |
| **Figure S3.** Scatterplot of the census tract-level prevalence of work-related and transportation-related noise exposure prevalence across US census tracts with working population >20 workers or total population >20 residents, respectively. |

|  |
| --- |
| **Figure S4.** Sensitivity analysis of model results presented in Figure 3 to test the robustness of the models after accounting for two indicators of socioeconomic status (the census tract-level percentage of low-income individuals and those without a high school diploma). |

1. Benjamin Roberts et al., “Imputation of Missing Values in a Large Job Exposure Matrix Using Hierarchical Information,” *Journal of Exposure Science & Environmental Epidemiology* 28, no. 6 (2018): 615–48, https://doi.org/10.1038/s41370-018-0037-x. [↑](#footnote-ref-1)
